# In Vitro–In Vivo Correlation in Dermal Delivery: The Role of Excipients

**DOI:** 10.3390/pharmaceutics13040542

**Published:** 2021-04-13

**Authors:** Avnish Patel, Fotis Iliopoulos, Peter J. Caspers, Gerwin J. Puppels, Majella E. Lane

**Affiliations:** 1 Department of Pharmaceutics, UCL School of Pharmacy, 29-39 Brunswick Square, London WC1N 1AX, UK; m.lane@ucl.ac.uk; 2 RiverD International B.V., Marconistraat 16, 3029 AK Rotterdam, The Netherlands; pcaspers@riverd.com (P.J.C.); gpuppels@riverd.com (G.J.P.); 3 Center for Optical Diagnostics and Therapy, Department of Dermatology, Erasmus MC, University Medical Center, 3015 CN Rotterdam, The Netherlands

**Keywords:** ibuprofen, excipients, skin delivery, Confocal Raman Spectroscopy, in vitro–in vivo correlation

## Abstract

The composition of topical and transdermal formulations is known to determine the rate and the extent of drug delivery to and through the skin. However, to date, the role of excipients in these formulations on skin delivery of actives has received little attention from scientists in the field. Monitoring skin absorption of both drug and vehicle may provide insights into the mechanism by which excipients promote permeation and may facilitate the design of effective and safer products. Previously, we have investigated the use of quantitative Confocal Raman Spectroscopy (CRS) to investigate the delivery of an active to the skin, and we also reported the first fully quantitative study that compared this method with the well-established in vitro permeation test (IVPT) model. To further explore the potential of quantitative CRS in assessing topical delivery, the present work investigated the effects of commonly used excipients on the percutaneous absorption of a model drug, ibuprofen (IBU). Permeation of IBU and selected solvents following finite dose applications to human skin was determined in vitro and in vivo by Franz diffusion studies and quantitative CRS, respectively. The solvents used were propylene glycol (PG), dipropylene glycol (DPG), tripropylene glycol (TPG), and polyethylene glycol 300 (PEG 300). Overall, the cumulative amounts of IBU that permeated at 24 h in vitro were similar for PG, DPG, and TPG (*p* > 0.05). These three vehicles outperformed PEG 300 (*p* < 0.05) in terms of drug delivery. Concerning the vehicles, the rank order for in vitro skin permeation was DPG ≥ PG > TPG, while PEG 300 did not permeate the skin. A linear relationship between maximum vehicle and IBU flux in vitro was found, with a correlation coefficient (R^2^) of 0.95. When comparing in vitro with in vivo data, a positive in vitro–in vivo (IVIV) correlation between the cumulative permeation of IBU in vitro and the total amount of IBU that penetrated the stratum corneum (SC) in vivo was observed, with a Pearson correlation coefficient (R^2^) of 0.90. A strong IVIV correlation, R^2^ = 0.82, was found following the linear regression of the cumulative number of solvents permeated in vitro and the corresponding skin uptake in vivo measured with CRS. This is the first study to correlate in vivo permeation of solvents measured by CRS with data obtained by in vitro diffusion studies. The IVIV correlations suggest that CRS is a powerful tool for profiling drug and vehicle delivery from dermal formulations. Future studies will examine additional excipients with varying physicochemical properties. Ultimately, these findings are expected to lead to new approaches for the design, evaluation, and optimization of formulations that target actives to and through the skin.

## 1. Introduction

Percutaneous delivery is an attractive route of drug administration, as it offers several advantages: It is non-invasive, thereby facilitating patient compliance, it is associated with reduced systemic adverse effects, it can achieve sustained drug release and constant levels of the drug in the plasma for a prolonged duration of action, and it avoids hepatic first pass metabolism [1]. However, the excellent barrier properties of human skin pose challenges to the development of efficacious formulations, thereby limiting the clinical uses of such products. Over the years, a range of vehicles and solvents have been examined for their potential to enhance percutaneous drug delivery.

To date, studies to assess the role of solvents on skin penetration of actives have included a range of biophysical methods, such as small and wide-angle X-ray diffraction studies, attenuated total reflectance-Fourier transform infrared spectroscopy (ATR-FTIR), and differential scanning calorimetry [2,3,4,5,6,7]. One of the most common methods to investigate vehicle effects on skin delivery of actives is in vitro permeation testing (IVPT) studies. Today, the IVPT model using human skin is accepted and recommended by a number of regulatory authorities for studying percutaneous absorption [8,9,10,11,12,13,14]. Results from IVTP have been shown to have excellent correlations with in vivo data [15]. However, most published IVPT studies in the literature examine solely the permeation of actives, while the fate of the excipients is often ignored. Previous reports from our group have monitored the skin delivery of both drug and excipients and have demonstrated co-transport of drugs with certain solvents [16,17,18]. More recently, Kung et al. [19] conducted IVPT studies to determine the delivery of methadone in binary and ternary vehicles and showed that the drug “tracked” the permeation of the examined solvent (Transcutol^®^ P) across the skin.

Confocal Raman spectroscopy (CRS) is a non-invasive optical method that combines Raman spectroscopy with a confocal signal detection scheme for high spatial resolution. CRS was first introduced as a tool in skin research by Caspers et al. [20] and is emerging as a powerful tool to study the distribution of substances in the skin in vitro and in vivo. Previous studies used CRS for the collection of qualitative information about the molecular composition of different skin layers [20], as well as for semi-quantitative measurements of endogenous ingredients [21] and topically applied substances across the skin in real-time [22,23,24]. Recently, we utilized a novel method to quantify CRS data, proposed by Caspers et al. [25], and we reported the first study to validate quantitative CRS measurements in vivo against IVPT data for determining percutaneous absorption of a model active, niacinamide [26]. An excellent in vitro–in vivo (IVIV) correlation (R^2^ = 0.98) was found following the linear regression of the cumulative amounts of the active permeated in vitro (μg/cm^2^) and the concentration of active inside the skin (mg/cm^3^) determined using CRS in vivo. A very good correlation between the cumulative permeation of the active in vitro and the total skin uptake per unit surface area (μg/cm^2^) in vivo was also observed (R^2^ = 0.94). These IVIV correlations support the use of quantitative CRS as a valid method for the measurement of actives delivered to the skin in vivo. A significant advantage of CRS is its capability for simultaneous profiling of actives and excipients in the tissue. However, to date, there are no published studies that have compared topical excipient uptake as measured with quantitative CRS in vivo with results from other validated methods, such as the IVPT model. Such correlations are important for quantitative CRS to be established as a valid technique to study permeation of excipients and to probe the mechanisms by which formulation components may affect percutaneous absorption.

The aims of the present work were, therefore, (i) to assess the potential use of quantitative CRS for studying the skin uptake of excipients in vivo and (ii) to probe any IVIV correlations between drug and/or excipient uptake. To this end, IVPT studies and in vivo CRS studies were performed to investigate the role of commonly used excipients on human skin delivery of a model drug, ibuprofen (IBU), from vehicles containing a volatile and a non-volatile component. The combination of volatile and non-volatile solvents has been previously described as a strategy to influence thermodynamic activity and increase dermal delivery [27,28]. Here, the volatile component was isopropyl alcohol (IPA), a commonly used excipient in topical products [29,30]. Permeation of IBU as well as PG, dipropylene glycol (DPG), tripropylene glycol (TPG), and polyethylene glycol 300 (PEG 300) following finite dose application of formulations to human skin was determined. The selection of these solvents was based on their wide use in dermal formulations and also on their reported potential to enhance permeation for IBU and other actives [31,32]. IBU was selected as a model active because it has been used in a wide range of marketed pharmaceutical formulations [33], and the ability of IBU to permeate the skin has been reported in previous studies [34,35].

## 2. Materials and Methods

### 2.1. Materials

IBU was a gift from Wyeth (Haversham, Hants, UK). PEG 300, PG, isopropyl alcohol (IPA), and trifluoroacetic acid (TFA) were supplied by Fisher Scientific (Leicestershire, UK). TPG, methanol, and High Performance Liquid Chromatography (HPLC) grade water were supplied by Sigma-Aldrich (Dorset, UK). DPG was supplied by Acros Organics (Fisher Scientific, Leicestershire, UK). Phosphate buffered saline (pH 7.4) was prepared using Dulbecco A tablets purchased from Oxoid Limited (Cheshire, UK). Excised abdominal human skin was obtained from the UK Human Tissue Bank and was stored at −20 °C until required (Research Ethics Committee reference 06/MRE04/37). The epidermis was separated by the heat separation method according to procedures reported previously [36].

### 2.2. HPLC Analysis

The HPLC analysis of IBU was performed using a HP1100 series (Hewlett-Packard, Palo Alto, CA, USA) as described previously [34]. Briefly, analysis was conducted at 35 °C using a Luna^®^ C_18_ column with dimensions 200 × 4.6 mm, 5 μm stationary phase, fitted with two C_18_ 4 × 3 mm, 5 μm SecurityGuard^TM^ cartridges (Phenomenex, Macclesfield, UK). The mobile phase consisted of methanol:water (80:20) with 0.1% (*v*/*v*) TFA and the flow rate was 1 mL/min. The sample injection volume was 15 µL, and the detection wavelength was 222 nm. The retention time of IBU under these conditions was 6 min. The HPLC method was validated according to the International Conference on Harmonisation (ICH) guidelines Q2, R1 [37]. The values for the limit of detection (LOD) and the limit of quantification (LOQ) were 0.1 µg/mL and 0.25 µg/mL, respectively.

### 2.3. Gas Chromatography (GC) Analysis

The analysis of solvents was performed using a 7890A GC system (Agilent Technologies, Santa Clara, CA, USA) equipped with a 10 µL autosampler and a flame ionization detector (FID). Two capillary columns were used for the selected solvents: A Zebron^TM^ ZB-WAX column (Phenomenex, Macclesfield, UK) with dimensions 30 m × 0.53 mm and film thickness of 1 µm and an Agilent J&W HP-5^®^ capillary column (Agilent Technologies, Santa Clara, CA, USA) with dimensions 30 m × 0.32 mm and film thickness of 0.25 µm. Nitrogen served as the carrier gas at a constant flow. For all methods, hydrogen and airflow were 35 and 350 mL/min, respectively. The details of the GC parameters for analysis of each solvent are given in Table 1. Chromatogram data were collected and integrated using Agilent ChemStation for GC systems (Agilent Technologies, Santa Clara, CA, USA).

All GC methods were validated according to the International Conference of Harmonization (ICH) guidelines Q2, R1 [37]. The accuracy value for all the solvents ranged between 95.9 and 103.5%. The RSD values of intra- and inter-day precision were ≤1.7% and 2.4%, respectively. The LOD values ranged from 0.3 to 1 μg/mL and the LOQ from 1 to 5 μg/mL.

### 2.4. Preparation of Formulations

The required mass of IBU was placed in a 7 mL glass vial with a screw cap, followed by the vehicle and finally IPA. The vehicles used were PG, DPG, TPG, and PEG 300. IBU was dissolved in the mixtures to produce liquid formulations (test solutions) of 5% (*w*/*w*). This concentration was selected as it has been commonly used in marketed preparations [33,38]. The formulations comprised differing amounts of IPA with either PG, DPG, TPG, or PEG 300. The IPA content for the formulations ranged from 79.4 to 87.0% (*w*/*w*). The amount of each solvent in the vehicles was selected thus that the IBU concentration in each residual phase was 80% of the drug saturated solubility, assuming complete evaporation of IPA. The compositions of the test solutions and details of the saturation solubility determinations are shown in Table 2.

### 2.5. Dynamic Vapor Sorption Studies

A dynamic vapor sorption (DVS) sorption apparatus (Surface Measurement Systems, London, UK) was used to monitor the change in mass of the formulations under controlled conditions. Quartz glass pans were connected to a microbalance, accurate to ± 0.0001 mg. Temperature and relative humidity (RH) were controlled throughout the experiment at 32 ± 1 °C and 50 ± 2% RH, respectively. 3.6 µL of samples were placed in the pan and data were recorded at 10 s intervals using DVSWin V3.01 (Surface Measurement Systems, London, UK) for 24 h.

### 2.6. In Vitro Finite Dose Permeation Studies

Finite dose human skin permeation studies were conducted using vertical glass Franz-diffusion cells as described previously [34], according to the Organization for Economic Co-operation and Development (OECD) guidelines [11,12]. The diffusion area was ~1 cm^2^, accurately measured for each Franz cell using an electronic digital micrometer (Fisher Scientific, Leicestershire, UK). The receptor compartment was filled with PBS pH 7.4 and 0.02% (*w*/*v*) sodium azide, in accordance with the FDA and OECD guidelines [11,12,39]. The experiments were conducted in a temperature-controlled water bath to ensure a skin surface temperature of 32 ± 1 °C, confirmed with a Digitron TM-22 Differential Digital Thermometer (RS Components, Corby, UK). Formulations were dosed at volumes of 3.6 μL in each Franz cell. Aliquots of 200 µL receptor fluid were sampled at the desired time points over 24 h and an equal volume of PBS solution was added to the receptor compartment. All the samples were analyzed using the validated HPLC method.

### 2.7. Confocal Raman Spectroscopy

In vivo human experiments were conducted using a Model 3510 SCA Skin Analyzer with RiverICon version 3.0.130327 software (RiverD International B.V., Rotterdam, The Netherlands). Two near-infrared (NIR) lasers were coupled to the instrument and emit monochromatic light, one at 785 nm, the other at 690 nm. Spectra in the fingerprint (400–1800 cm^−1^) and high wavenumber region (2500–4000 cm^−1^), respectively, were recorded. On the day of the experiment, the instrument was calibrated using an external National Institute of Standards and Technology (NIST) glass calibration standard and the instrument’s internal Raman calibration standards (a neon lamp and a polymethylmethacrylate Raman sample). Calibration was considered successful when it passed the built-in criteria in the software and when the signal-to-noise ratio was above 30. The reference spectrum of each compound was calculated as the average of 10 frames taken sequentially, with a 30 s exposure time. For IBU, 200 mg were dissolved in 300 mg of Transcutol^®^ P, because IBU has been reported to be readily soluble in this solvent [40]. Subsequently, the reference spectrum of IBU was obtained following subtraction of the solvent spectrum.

For the measurements, an application site measuring (4 × 4) cm^2^ was delineated on the volar forearm of 8 volunteers (6 males, 2 females, age range 26–32). Institutional ethical approval was obtained prior to the recruitment of volunteers for this study (Reference Number 0234/0624). A finite dose, 40 µL, of the formulation was applied evenly over the marked area (16 cm^2^), without rubbing. On another skin site, a series of scans were taken with the fingerprint region laser, which served as control scans. The treated areas were measured 40 min post formulation application with the 785 nm laser. Control and formulation scans used a 10 s exposure time with 2 µm steps to a final depth of 30 µm; a minimum of 4 and 8 scans were used, respectively. Skin water profiles were also measured using the 671 nm high wavenumber laser. The water concentration profiles were taken to allow the calculation of SC thickness, according to procedures described previously [26]. A 0.5 s exposure time was used with 2 µm steps to a final depth of 40 µm.

### 2.8. Data Analysis

The data were collated and processed using Microsoft^®^ Excel 2010 software (Microsoft Corporation, Redmond, WA, USA) and GraphPad Prism Statistics software (GraphPad Software LLC, version 9.0.1, San Diego, CA, USA). The Raman spectra were analyzed using SkinTools 3 (v. 3.3.20200720, RiverD International B.V., Rotterdam, The Netherlands) software. One-way analysis of variance (ANOVA) was used to compare the means of different groups. Post hoc multiple comparisons were performed using the Tukey test. A probability of *p* < 0.05 was considered to be statistically significant. All results, unless stated otherwise, were presented as mean ± standard deviation (SD). All statistical analyses were conducted using GraphPad Prism Statistics software (GraphPad Software LLC, version 9.0.1, San Diego, CA, USA). The correlations were plotted as mean ± SD and were calculated using Pearson’s correlation coefficient (R^2^).

## 3. Results and Discussion

### 3.1. Dynamic Vapour Sorption Studies

**Figure 1:**
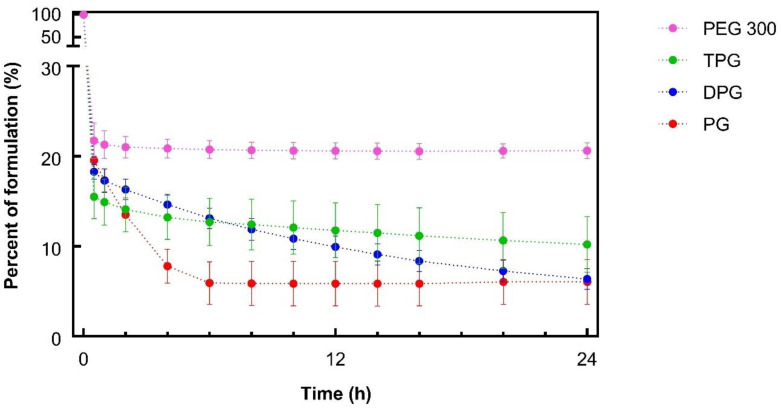
Percentage weight remaining for ibuprofen (IBU) formulations over 24 h under controlled conditions, 32 ± 1 °C and 50 ± 2% RH (mean ± SD; *n* = 3).

Figure 1 shows the DVS results over 24 h. A rapid weight loss for all samples was evident, which may be attributed to the evaporation of IPA. Neat IPA (3.6 μL) was found to evaporate completely within 1 min when monitored under the same conditions (32 °C and 50% RH, Figure S1). The glycol solvents PG, DPG, and TPG, were also volatile, and they had similar percent mass recoveries at 24 h, i.e., 6.1, 6.4, and 10.2%, respectively (*p* > 0.05).

With regards to PEG 300 containing samples, approximately 20% of the applied mass remained from 1 h onwards, and this value was greater than the other vehicles (*p* < 0.05). Given that 20% (*w*/*w*) of the sample corresponded to IBU and PEG 300 (Table 2), the data indicated that PEG 300 did not evaporate over the course of the study. The volatility of neat PG and DPG under the same conditions of temperature and humidity was previously reported by Haque et al. [16]. To our knowledge, the evaporation of TPG and PEG 300 had not been previously investigated using DVS studies. The sorption behavior of different molecular weights of PEG (i.e., PEG 200 and PEG 400) has been examined, and no evaporation has been reported for these compounds [41,42,43].

### 3.2. In Vitro Finite Dose Permeation and Mass Balance Studies

#### 3.2.1. Skin Permeation of IBU

**Figure 2:**
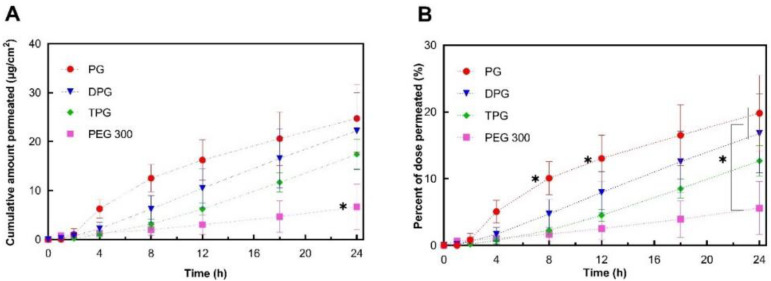
Permeation profiles of IBU in human epidermis after application of 5% IBU formulations expressed as (**A**) cumulative amounts (μg/cm^2^) and (**B**) percentages (%) of the dose applied (mean ± SD; 5 ≤ *n* ≤ 6, * *p* < 0.05).

The permeation profiles of IBU in human skin in vitro from the various test solutions are shown in Figure 2.

**Figure 3:**
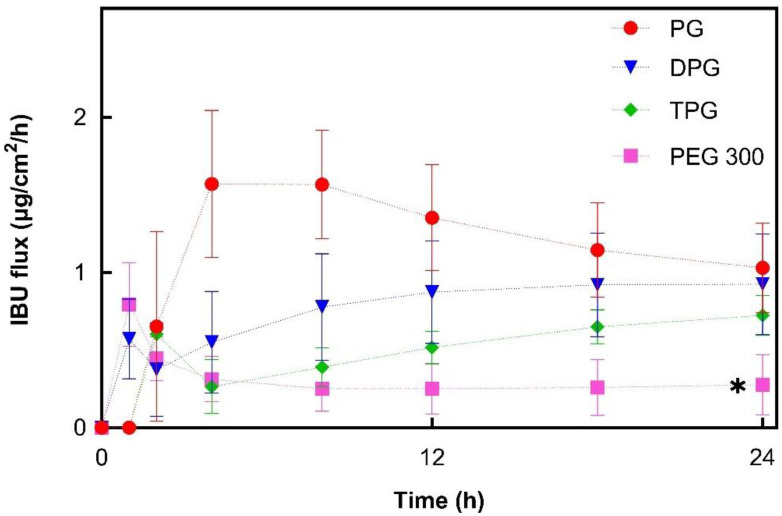
Estimated flux profiles of IBU through human epidermis after application of 5% IBU samples (mean ± SD; 5 ≤ *n* ≤ 6, * *p* < 0.05).

The cumulative amounts of IBU that permeated through human skin at 24 h for PG (24.7 μg/cm^2^), DPG (22.2 μg/cm^2^), and TPG (17.4 μg/cm^2^) were significantly higher (*p* < 0.05) than for PEG 300 (6.7 μg/cm^2^). The PG vehicle delivered a significantly (*p* < 0.05) higher percentage of the IBU dose than all other vehicles at 8 h (10.1% of the dose applied) and at 12 h (13.0% of the dose applied). At 24 h, both PG and DPG samples delivered a significantly higher percent of IBU when compared to PEG 300 (*p* < 0.05). The performance of several commercial IBU topical formulations in terms of promoting drug delivery has previously been reported by Hadgraft et al. [33]. These researchers conducted IVPT studies under finite dose conditions (5 mg/cm^2^) over 48 h and evaluated the permeation of IBU from a range of commercial products containing 5% IBU. For all commercial formulations examined, percentage permeation values did not exceed 10% of the IBU applied at 24 h, although the exact values were not given by the authors for this time-point. The IBU flux profiles (μg/cm^2^/h) were determined by dividing the cumulative amount of IBU permeated in a given sampling interval by the length of that interval in hours [9]. Results are shown in Figure 3.

For PG, the IBU flux within the first 4 h increased to a maximum flux of 1.6 μg/cm^2^/h. From this point, it began to decline, suggesting that the formulation could not maintain delivery of IBU over time. This may be attributed to the depletion of PG from the formulation. The DPG sample showed an increasing IBU flux up to 18 h (0.9 μg/cm^2^/h) and flux was subsequently relatively constant. For TPG the flux increased over the 24 h period, indicating that no depletion occurred. IBU flux profiles for PEG 300 showed a rapid increase within the first hour (up to 0.8 μg/cm^2^/h) followed by a successive reduction to a relatively constant flux from 8 to 24 h (0.3 μg/cm^2^/h). Overall, PG, DPG, and TPG produced significantly higher IBU fluxes at 24 h compared to the PEG 300 formulation (*p *< 0.05). The lower IBU flux from pure PEG 300 may reflect the lower degree of volatility found for this formulation by the DVS experiments (Figure 1). Oliveira, Hadgraft, and Lane [28] studied the influence of volatile formulations on drug transport using the IVPT model with human skin and underlined the potential of volatile components to optimize dermal delivery. Evaporation of the volatile vehicle components from the skin surface may increase the thermodynamic activity, and subsequently, the driving force of drug permeation. The initial spike in IBU flux in the first 1 h may be attributed to IPA’s rapid evaporation, while the residual vehicle, namely PEG 300, appears to have low efficacy in promoting IBU permeation. PEG 300 has been used as a solvent in topical formulations; however, its effect on promoting permeation of compounds has not been investigated in human skin. The influence of PEGs of different molecular weights (i.e., PEG 200 and PEG 400) has been examined in the literature, and limited efficacy in promoting permeation has generally been reported. Intarakumhaeng and Li [44] examined the delivery of corticosterone to human skin from several solvents under finite dose conditions (10 μL). These authors reported that the amounts of drug permeated across the skin from PEG 400 (0.4% of the dose applied) were the lowest compared with all other solvents (7–15% of the dose applied). Zhang et al. [41,45] reported no permeation or skin retention of niacinamide from a neat PEG 400 solution. Combinations of PEG 400 with solvents that promoted niacinamide delivery as neat vehicles, i.e., PG, dimethyl isosorbide or Transcutol^®^ P, resulted in lower percentage permeation of the active compared with the corresponding single solvent systems (*p* < 0.05). Here, all vehicles outperformed PEG 300 in promoting IBU delivery across the skin over 24 h (*p* < 0.05).

#### 3.2.2. Skin Permeation of the Vehicle

**Figure 4:**
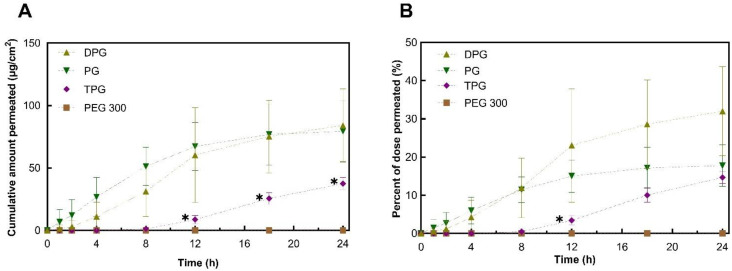
Permeation profiles of vehicles in human epidermis after application of samples expressed as (**A**) cumulative amounts (μg/cm^2^) and (**B**) percentages (%) of dose applied (mean ± SD, *n* = 5, * *p* < 0.05).

To investigate further the roles of the vehicles, the skin permeation of the excipients was determined. Permeation results for PG, DPG, TPG, and PEG 300 are shown in Figure 4.

**Figure 5:**
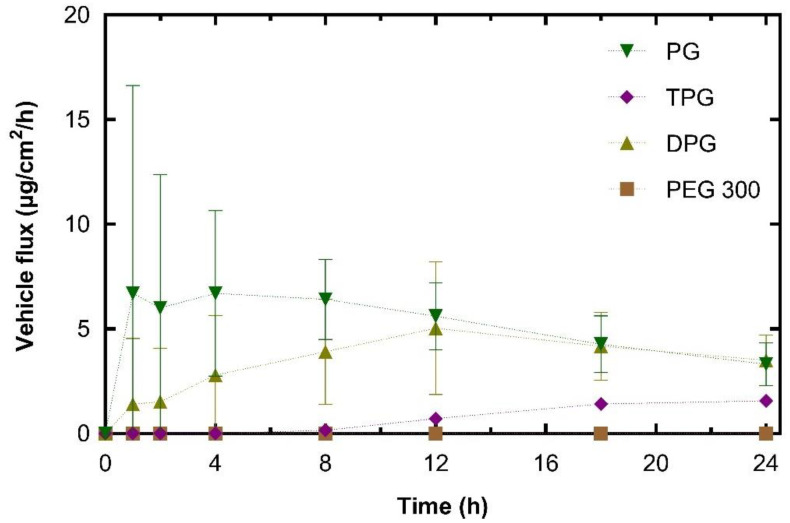
Estimated flux profiles for vehicles through human epidermis after application of samples (mean ± SD, *n* = 5).

The plateaus observed for the permeation profiles for PG and DPG were indicative of vehicle depletion. The depletion of the vehicle from the residual phase resulted in the reduction of solvent transport over the 24 h period. The vehicle can be depleted by evaporation to the environment and by skin uptake. The DVS results (Figure 1) demonstrated that the PG, DPG, and TPG would evaporate over 24 h. The lack of vehicle in the skin may result in IBU crystallization on and in the membrane [46,47,48]. Conversely, the presence of a vehicle inside the skin may enable IBU to remain in solution and sustain drug transport. A plateau was reached for PG after 12 h, and a plateau appears to emerge for DPG after 18 h. Only after 8 h did TPG penetrate the membrane, after which permeation seemed to be relatively steady. Statistical analysis of the cumulative amounts of vehicles permeated showed no differences between the permeation of PG and DPG at 8, 12, or 24 h (*p* > 0.05). Figure 4 shows that significantly more PG and DPG permeated at 8, 12, and 24 h when compared to TPG (*p* < 0.05). With regards to PEG 300, this solvent did not permeate the human epidermis over 24 h. The vehicle flux profiles are shown in Figure 5.

The peak PG flux obtained here occurred before that of IBU. This was also the case for DPG, where vehicle flux peaked at 12 h and IBU flux at 18 h. The maximum flux value for PG (10.8 μg/cm^2^/h) was significantly higher than TPG (1.9 μg/cm^2^/h) (*p* < 0.05). There was no significant difference in the maximum vehicle flux between DPG (5.2 μg/cm^2^/h) and TPG (*p* > 0.05). In the literature, there are limited published data on the dermal penetration and flux values of these solvents. Fasano et al. [49] conducted infinite dose studies (application of 1.2 mL/cm^2^) using human abdominal tissue to measure the permeation of PG and DPG. Under these conditions, there was no difference between the amount of PG or DPG that permeated for at least 10 h. These authors also reported that PG had a higher steady-state flux than DPG, 97.6 and 39.3 μg/cm^2^/h, respectively. More recently, Thombre et al. [50] conducted IVPT studies in human skin and measured the permeation of neat PG following an application dose of 10 mg/cm^2^. These researchers reported a pseudo-steady state flux value of 44.20 μg/cm^2^/h, which was achieved between the 4 and 24 h time points. It should be noted, however, that in the present study, the amounts of PG that were applied to the skin are considerably lower than previous reports. Additionally, the shapes of the permeation profiles of vehicles indicate a depletion of the solvent’s PG and DPG from the skin.

The percentage permeation of PG over 24 h was 17.7%. These data were similar to the findings for the permeation of PG across human skin reported in previous studies. Haque, et al. [16] reported that 21.9% of the applied PG had permeated at 48 h, and Thombre, et al. [50] found that percent permeation at 24 h was 13.9% of the applied dose. A significantly higher percentage of PG and DPG, 11% for both, permeated at 8 h when compared to TPG (3.4%, *p* < 0.05), while no PEG 300 had permeated at this time point. At 12 h the percentages of PG and TPG permeation were not significantly different; however, a higher percent of DPG, 30%, had permeated when compared to TPG, 15% (*p* < 0.05). The percent permeation of DPG found here (32.0%) was greater than the value reported by Haque, Rahman, Thurston, Hadgraft, and Lane [16], which was 8.0%. This difference is likely to be attributed to (i) the presence of the penetration enhancer IPA in the formulations or (ii) different interactions of DPG with IBU compared with the drug used by Haque, et al. [16], i.e., anthramycin. As regards the absorption of PEG 300, no permeation was found for this solvent at 24 h.

#### 3.2.3. IBU and Vehicle Flux Correlation

**Figure 6:**
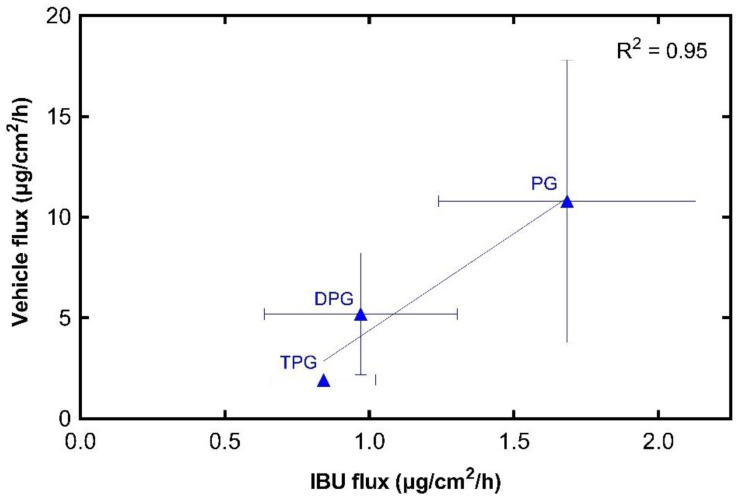
Correlation of maximum vehicle flux and maximum IBU flux through human epidermis (mean ± SD, *n* = 5).

Figure 6 shows that the permeation of IBU from every vehicle was dependent on the permeation of the corresponding excipient. The results indicate a strong positive relationship between maximum vehicle flux and maximum IBU flux, with a Pearson correlation coefficient (R^2^) of 0.95. The glycol vehicles (i.e., PG, DPG, TPG) permeated the skin, while no amounts of PEG 300 were detected in the receptor fluid at 24 h. Diffusion of the vehicle into and through the skin can promote IBU partitioning to the membrane.

Of the solvents selected, PG has been studied for many years [51,52,53]; however, the exact mechanism by which PG may affect the permeation of actives is not fully established. Zhang et al. [54] used various PG:water systems to examine the permeation of a series of phenolic compounds in the human epidermis under infinite doses conditions in vitro. It was found that the maximum flux of the actives increased with increasing percentages of PG in the vehicles. The solvent uptake into the trypsin-separated SC was also determined for every vehicle. These researchers proposed that the permeation of the phenolic compounds was enhanced by the uptake of PG into the SC. In a different study, Trottet et al. [55] applied finite doses, 10 mg/cm^2^, of loperamide formulations to human skin to determine the solvent effects on drug delivery. These authors reported a correlation between the amount of PG dosed on the skin and the amount of drug that permeated, and they proposed that the decline in the rate of drug permeation was due to the depletion of PG over time. Watkinson et al. [56] investigated the effects of PG on IBU permeation in human epidermis from saturated solutions in vitro. The solutions tested were mixtures of PG and water at various volume ratios, as well as neat PG and neat water. These researchers reported that the permeation rate of IBU increased with an increasing percentage of PG in the mixtures, and they suggested that the influence of PG in the PG:water systems was primarily on the solubility and partitioning behavior of IBU. More recently, Haque et al. [16,17] examined the delivery of anthramycin and solvents in human epidermis from various vehicles. These authors reported that permeation of anthramycin was associated with high amounts of PG in the receptor phase, and they suggested that the drug “tracked” the permeation of PG across the skin. Co-transport of anthramycin and the solvent DPG was also reported. In a recent study by our group [19], the hydrophilic solvent Transcutol^®^ P, was found to drive the permeation of methadone across human skin. Permeation of methadone had an excellent correlation with the permeation of the solvent (R^2^ = 0.97). The solvent TPG is less studied; however, it is hydrophilic, and PG may promote permeation by increasing the partition or solubility of a solute in the skin.

### 3.3. In Vivo Finite Dose Permeation Studies—Confocal Raman Spectroscopy

#### Skin Permeation of IBU and Vehicles

**Figure 7:**
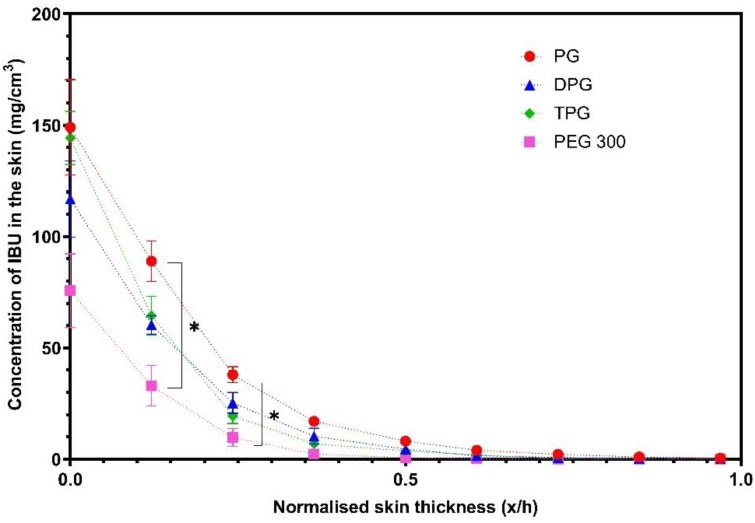
Concentration profiles of IBU (mg/cm^3^) in the *stratum corneum* (SC) after application of various formulations (mean ± SEM, 4 ≤ *n* ≤ 5, * *p* < 0.05).

IBU in the SC was measured 40 min post-application of formulations (Figure 7).

**Figure 8:**
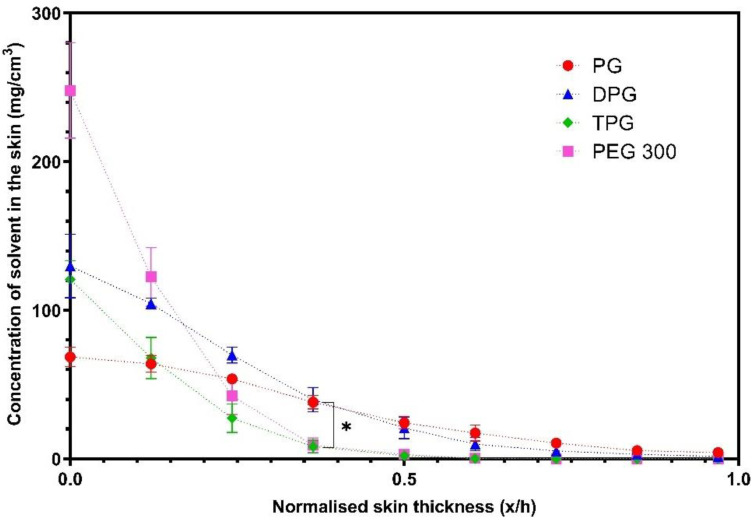
Concentration profiles of vehicles (mg/cm^3^) in the SC after application of various vehicles (mean ± SEM, 4 ≤ *n* ≤ 5, * *p* < 0.05).

Figure 7 shows that PG delivered significantly higher amounts of IBU at 0.2 and 0.3 x/h depth intervals when compared to the PEG 300 formulation (*p* < 0.05). No significant difference was found between the other vehicles (*p* > 0.05). The natural variation between the human volunteers that were recruited may explain the lack of significant differences between the PG, DPG, and TPG formulations. The vehicle concentration profiles in the SC are shown in Figure 8.

The solvents PG and DPG, penetrated across the SC thickness, while the TPG and PEG 300 were not found deeper than the 0.5 x/h SC depth interval. The amounts of PG and DPG that penetrated to 0.4 x/h depth were significantly higher compared with either TPG or PEG 300 (*p* < 0.05). The concentration of PG at 0.5 x/h was found to be significantly higher than TPG and PEG 300 (*p* < 0.05). The findings indicate that PG and DPG permeated the human epidermis more effectively than TPG and PEG 300, and these results were consistent with the rank order of solvent penetration found in vitro. The greater skin uptake of PG in the SC may explain the efficiency of this solvent to deliver IBU, also found in the in vitro studies. The solvent and drug transport have been examined in previous studies using CRS. Pudney et al. [57] utilized CRS to monitor the permeation of trans-retinol in human skin in vivo from two vehicles: A mixture of propylene glycol (PG) with ethanol and a single solvent system comprising of caprylic/capric acid triglycerides. The penetration of active and solvents was determined for up to 10 h after application of finite doses (70 μL/16 cm^2^) of formulations. These researchers reported that PG penetrated the SC, unlike caprylic/capric acid triglycerides that remained on the surface or in the uppermost SC (top 8 μm). Additionally, PG/Ethanol formulations outperformed caprylic/capric acid triglycerides in terms of promoting penetration of the active, with trans-retinol penetration being correlated with the depth of penetration of PG. In a later study by the same group, Melot et al. [58] used the same experimental protocol to examine the effects of the CPE oleic acid and the surfactant Triton X-100 on the permeation of trans-retinol. Either solvent was added to caprylic/capric acid triglycerides, in which the active was highly soluble. Greater penetration of trans-retinol was found when Triton X-100 or oleic acid were used compared with the neat caprylic/capric acid triglycerides, which alone did not promote permeation of the active. In addition, these researchers reported that although the neat solvent caprylic/capric acid triglycerides remained on the skin surface, the addition of either Triton X-100 or oleic acid resulted in this solvent penetrating inside the SC, to a depth below 8 μm from the skin surface. Mohammed et al. [59] later investigated the percutaneous absorption of niacinamide following a 30 min application of various formulations in vivo. The depth profiles of solvents were also obtained. A positive linear correlation (R^2^ = 0.99) between the signal intensity of the active and of certain solvents inside the skin, i.e., PG and dimethyl isosorbide was reported, indicating co-transport of niacinamide and these solvents.

### 3.4. In Vitro–In Vivo Correlations

**Figure 9:**
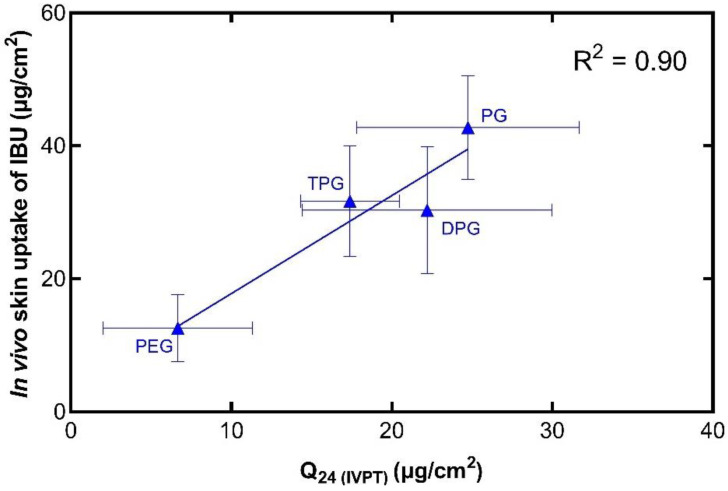
Correlation of the in vitro cumulative amount of IBU permeated after 24 h (mean ± SD, *n* = 5) and the total amount of IBU in the SC per skin surface area in vivo (mean ± SD, 4 ≤ *n* ≤ 5).

Previously, we reported that results for drug permeation obtained from CRS measurements correlated with findings from in vitro diffusion studies in human skin [26,59]. The concept of IVIV correlation has been an important focus for the pharmaceutical industry and the regulatory bodies [10]. Clearly, such correlation would be a useful aid for the development, optimization, and evaluation of formulations. Here, an attempt is made to correlate the delivery of IBU in vitro with IBU skin uptake in vivo. For this, the total amount of drug (μg) per unit area (cm^2^) that penetrated the SC in vivo was plotted against the corresponding in vitro cumulative permeation, as described in our previous work [26]. Results are shown in Figure 9.

**Figure 10:**
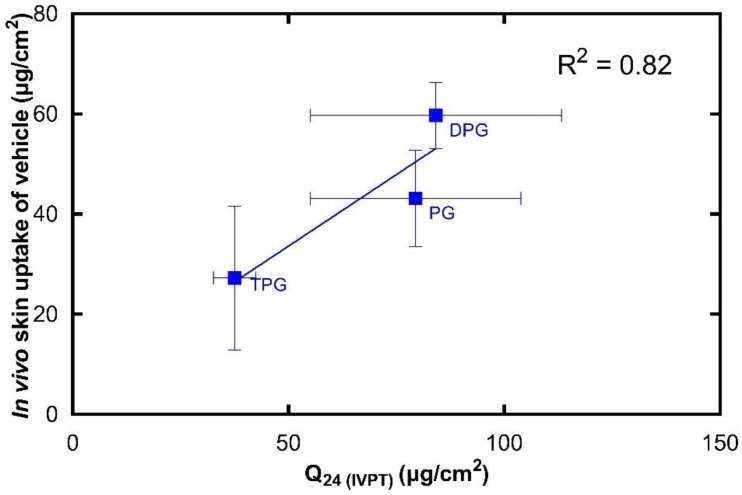
Correlation of the in vitro cumulative amount of vehicle permeated after 24 h (mean ± SD, *n* = 5) and the total amount of vehicle in the SC per skin surface area in vivo (mean ± SD, 4 ≤ *n* ≤ 5).

The in vivo and in vitro results show that there is a positive correlation (R^2^ = 0.90) between the two sets of experiments. Additionally, this relationship has been demonstrated with the use of finite doses in both instances, i.e., with doses that would be used clinically. Comparing these results with the in vitro Franz cell permeation studies using human skin, there are some similarities in the performance of the formulations. The vehicle containing PG outperformed the other vehicles in promoting IBU delivery, as found in the in vivo CRS experiments and in the first 8 h of in vitro permeation. In addition, among the vehicles, PEG 300 delivered the lowest amounts of IBU in vitro, and this is consistent with the low amounts of IBU found in the SC in vivo. The IVIV correlation of the solvents that were found to permeate the skin in vitro was also carried out, as shown in Figure 10.

There is a strong linear correlation (R^2^ = 0.82) between the in vivo skin uptake and in vitro cumulative permeation of the vehicle. To our knowledge, there is no previous work that has correlated CRS data on vehicle permeation with corresponding in vitro diffusion findings. It should be mentioned that human skin is known to have high levels of barrier variability [39]. The natural biological variation for both in vitro and in vivo studies may be reflected in the SD values observed in Figure 9 and Figure 10. We previously published the first study to validate the novel quantitative CRS method against the well-established IVPT, where the in vitro permeation of a model active through human skin was positively correlated with the in vivo skin uptake. Here, an excellent correlation is also found between the in vitro permeation and in vivo skin uptake of solvents. Measuring the absorption of solvents in the SC is important for understanding the effects of excipients on percutaneous absorption and should also provide useful insights into formulation efficacy. Additionally, this information will be useful for safety and toxicological studies as it may allow better risk assessment where pharmaceutical formulations and other preparations are applied to the skin.

## 4. Conclusions

The present work examined the effect of four solvents on IBU skin delivery in human skin in vitro and in vivo. This is the first study to correlate vehicle uptake to the skin measured with quantitative CRS in vivo, with data obtained by a well-established in vitro method, i.e., the IVPT model. The rank order of permeation of vehicles was DPG ≥ PG > TPG, while no permeation was found for PEG 300. The permeation of IBU through human skin in vitro was found to be highly dependent on the permeation of the vehicle, with a correlation coefficient (R^2^) of 0.95. When comparing the in vitro and in vivo data, excellent correlations were found between the cumulative permeation of drug and vehicle (R^2^ = 0.90 and 0.82, respectively) in vitro and the corresponding total amount taken up in the SC in vivo. The IVIV correlations indicate that CRS is a powerful tool for profiling drug and vehicle delivery from dermal formulations. The findings support the use of quantitative CRS as a non-invasive method to evaluate skin delivery. This will aid in the development, optimization, and assessment of formulations and minimize the need for time-consuming and expensive clinical endpoint studies. Future studies will examine additional excipients with varying physicochemical properties. The IVIV correlations will be further explored in terms of solvent and drug uptake, with a wider range of drugs and vehicles.

## Figures and Tables

**Table 1:** Gas chromatography (GC) analytical parameters for solvents.

Solvent	PG	DPG	TPG	PEG 300
**Column**	ZB-WAX	ZB-WAX	ZB-WAX	HP-5^®^
**Injection Volume (μL)**	0.5	0.5	0.5	2
**Split Ratio**	1:1	1:1	1:1	6:1
**Septum Purge Flow (mL/min)**	3	3	3	3
**Inlet Temperature (°C)**	225	225	225	250
**Detector Temperature (°C)**	300	300	300	300
**Oven Conditions**	80	80	80	80
	1	1	1	1
	200	200	250	300
	15	15	40	50
	0	0	2.25	2
**Retention Time of Analyte (min)**	5.3	7	4.3	4.2

**Table 2:** Quantities of formulation components expressed as % (*w*/*w*).

Formulation	Vehicle	IBU	IPA
**PG**	15.6	5.0	79.4
**DPG**	10.7	5.0	84.3
**TPG**	8.0	5.0	87.0
**PEG 300**	15.3	5.0	79.7

## Data Availability

The data presented in this study are available on request from the corresponding author.

## Supplementary Materials

The following are available online at https://www.mdpi.com/article/10.3390/pharmaceutics13040542/s1, Figure S1: Percent of weight loss of IPA under static conditions 50% RH and 32 °C after application of 3.6 µL on a quartz glass pan (mean ± SD; *n* = 3), Figure S2. Replicate data points of in vitro cumulative permeation after 24 h (*n* = 5) against the corresponding mean total amount in the SC per skin surface area in vivo (4 ≤ *n* ≤ 5). Table S1: Saturation solubility of IBU in neat solvents at 32 ± 1 °C (mean ± SD; *n* = 5).
