# Supplementary material for: In Vitro–In Vivo Correlation in Dermal Delivery: The Role of Excipients
Source: Pharmaceutics. 2021 Apr 13;13(4):542. doi: 10.3390/pharmaceutics13040542 (PMC8069833; doi:10.3390/pharmaceutics13040542)
Supplement: Supplementary file 1 [file pharmaceutics-13-00542-s001.pdf]

pharmaceutics

Supplementary Materials: In Vitro–In Vivo Correlation in Dermal Delivery: The Role of Excipients

Avnish Patel [1,*], Fotis Iliopoulos [1,*], Peter J. Caspers [2,3], Gerwin J. Puppels [2,3] and Majella E. Lane [1]

Solubility of IBU in vehicles

An excess of IBU was placed in 2 mL Eppendorf® tubes in addition to 1 mL of the appropriate vehicle. Tubes were sealed with Parafilm M® and placed on a rotary mixer (Stuart, UK) in a thermostatic oven (Jouan, France) held at 32 ± 1 °C for 48 h. Tubes were checked periodically to ensure an excess of IBU was present. After 48 h tubes were centrifuged at 13,200 rpm for 15 min. The supernatant was removed and samples were centrifuged again at 13,200 rpm for 15 min. All samples were diluted were analysed by HPLC. The saturation solubility values of IBU in the solvents investigated are shown in Table S1. The solubility values for IBU in the solvents were used for the development of formulations.

Table S1. Saturation solubility of IBU in neat solvents at 32 ± 1 °C (mean ± SD; $n = 5$).

	Saturation Solubility of IBU (mg/mL)
**TPG**	453.8 ± 5.7
**DPG**	382.0 ± 2.5
**PEG 300**	324.4 ± 2.3
**PG**	296.0 ± 5.0

Dynamic vapour sorption studies of neat IPA

DVS results for 3.6 μL of neat IPA, and ethanol for comparative purposes, show complete evaporation for both alcohols with a faster evaporation rate observed with IPA.

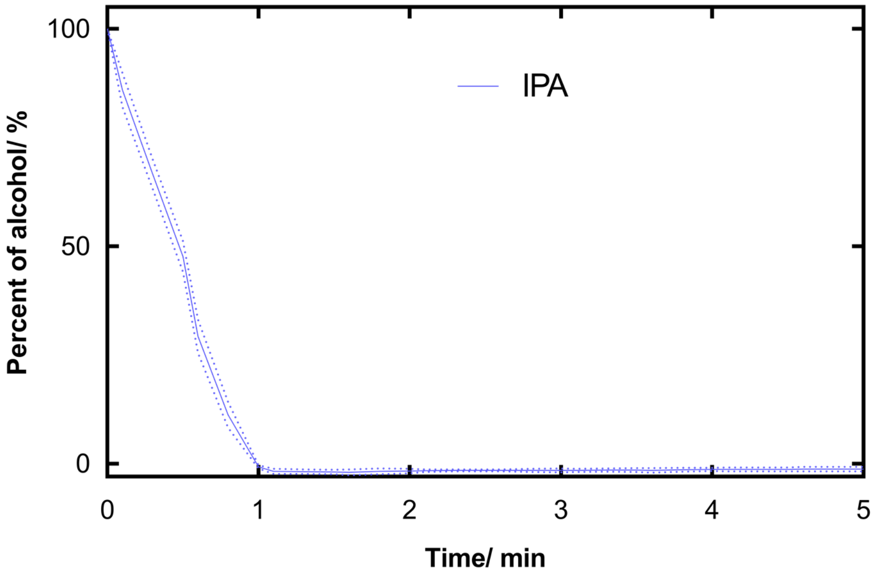

Figure S1. Percent of weight loss of IPA under static conditions 50% RH and 32 °C after application of 3.6 μL on a quartz glass pan (mean ± SD; $n = 3$).

Individual replicates

The Figure S2 shows the replicate data points for the in vitro cumulative permeation of either IBU or vehicle against the corresponding in vivo skin uptake. The apparent spread of the replicates is indicative of the biological variation of human skin.

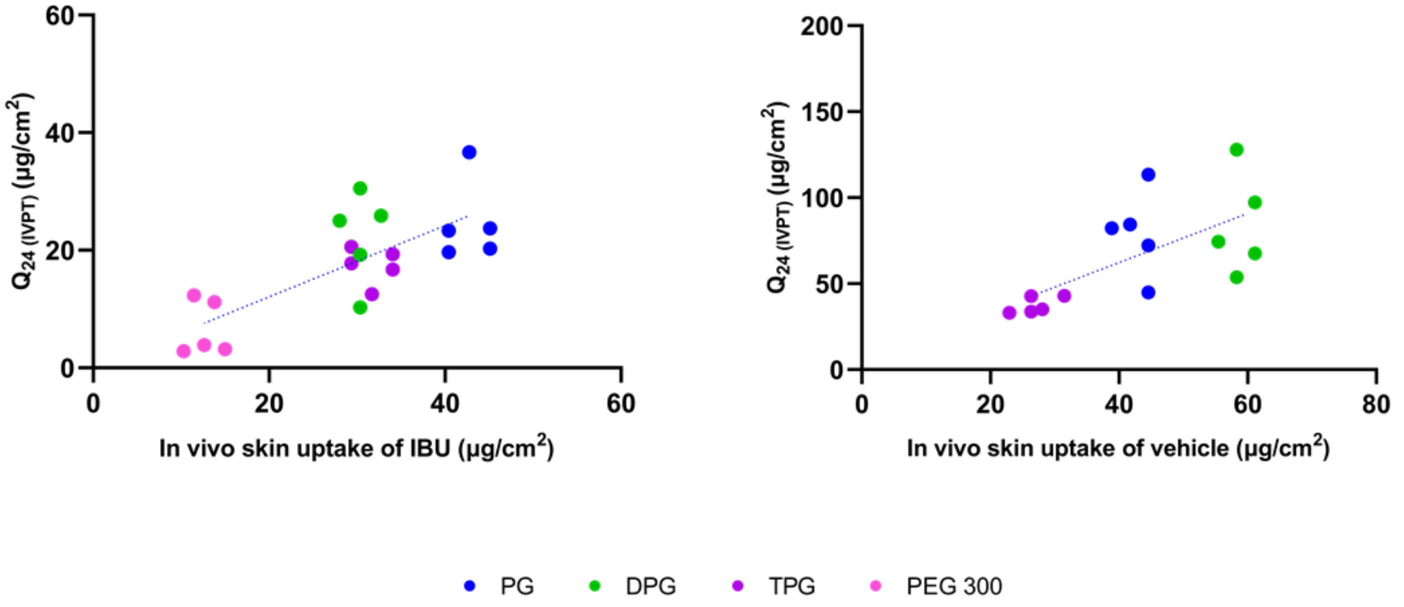

Figure S2. Replicate data points of in vitro cumulative permeation after 24 h (n = 5) against the corresponding mean total amount in the SC per skin surface area in vivo ($4 \leq n \leq 5$). Plots are staggered to represent data distribution and minimise overlap of individual data points.
